# Comparison of Endoscopic and Artificial Intelligence Diagnoses for Predicting the Histological Healing of Ulcerative Colitis in a Real-World Clinical Setting

**DOI:** 10.1093/crocol/otae005

**Published:** 2024-01-20

**Authors:** Teppei Omori, Tomoko Yamamoto, Shun Murasugi, Miki Koroku, Maria Yonezawa, Kouichi Nonaka, Yoji Nagashima, Shinichi Nakamura, Katsutoshi Tokushige

**Affiliations:** Institute of Gastroenterology, Tokyo Women’s Medical University, Tokyo, Japan; Department of Surgical Pathology, Tokyo Women’s Medical University, Tokyo, Japan; Institute of Gastroenterology, Tokyo Women’s Medical University, Tokyo, Japan; Institute of Gastroenterology, Tokyo Women’s Medical University, Tokyo, Japan; Institute of Gastroenterology, Tokyo Women’s Medical University, Tokyo, Japan; Department of Digestive Endoscopy, Tokyo Women’s Medical University Hospital, Tokyo, Japan; Department of Surgical Pathology, Tokyo Women’s Medical University, Tokyo, Japan; Institute of Gastroenterology, Tokyo Women’s Medical University, Tokyo, Japan; Institute of Gastroenterology, Tokyo Women’s Medical University, Tokyo, Japan

**Keywords:** artificial intelligence, endocytoscopy, Mayo endoscopic score, ulcerative colitis

## Abstract

**Background:**

Artificial intelligence (AI)-assisted colonoscopy systems with contact microscopy capabilities have been reported previously; however, no studies regarding the clinical use of a commercially available system in patients with ulcerative colitis (UC) have been reported. In this study, the diagnostic performance of an AI-assisted ultra-magnifying colonoscopy system for histological healing was compared with that of conventional light non-magnifying endoscopic evaluation in patients with UC.

**Methods:**

The data of 52 patients with UC were retrospectively analyzed. The Mayo endoscopic score (MES) was determined by 3 endoscopists. Using the AI system, healing of the same spot assessed via MES was defined as a predicted Geboes score (GS) < 3.1. The GS was then determined using pathology specimens from the same site.

**Results:**

A total of 191 sites were evaluated, including 159 with a GS < 3.1. The MES diagnosis identified 130 sites as MES0. A total of 120 sites were determined to have healed based on AI. The sensitivity, specificity, positive predictive value (PPV), negative predictive value (NPV), and accuracy of MES0 for the diagnosis of GS < 3.1 were 79.2%, 90.6%, 97.7%, 46.8%, and 81.2%, respectively. The AI system performed similarly to MES for the diagnosis of GS < 3.1: sensitivity, 74.2%; specificity: 93.8%; PPV: 98.3%; NPV: 42.3%; and accuracy: 77.5%. The AI system also significantly identified a GS of < 3.1 in the setting of MES1 (*P* = .0169).

**Conclusions:**

The histological diagnostic yield the MES- and AI-assisted diagnoses was comparable. Healing decisions using AI may avoid the need for histological examinations.

## Introduction

Ulcerative colitis (UC) is a refractory intestinal disorder caused by a combination of mechanisms, including immunological mechanisms.^1^ The number of patients with UC in Japan is increasing.^2^ Several patients with UC have recurrent or chronic persistent intestinal inflammation. Continued intestinal inflammation decreases the patient’s quality of life and results in hospitalization and the development of intestinal inflammation-related cancers.^3–5^

Achieving endoscopic mucosal remission is a therapeutic goal for UC that can help avoid these complications. The Selecting Therapeutic Targets in Inflammatory Bowel Disease-II initiative, proposed by the International Organization for the Study of Inflammatory Bowel Disease, recently recommended that endoscopic remission be a therapeutic goal to achieve the higher goals of improved quality of life and disappearance of disability.^6^ The Mayo endoscopic score (MES) is used in the endoscopic evaluation of UC.^7,8^ Endoscopic mucosal remission is often defined by an MES of 0 or 1.^9,10^ However, endoscopic assessment involves a subjective component and is prone to variability.^8,11,12^ Histologic healing has been reported as a more advanced therapeutic goal for UC^13–16^ and has the potential to demonstrate mucosal healing more objectively than an endoscopic evaluation. However, the determination of histologic healing requires invasive biopsies.

The EndoBRAIN-UC system (Cybernet Systems) is a fully automated diagnostic system with artificial intelligence (AI) that uses endocytoscopy to identify the presence of histologic inflammation associated with UC. The AI system analyzes features such as invisibility, dilation, and hyperplasia of capillaries in the colonic mucosa via ultra-magnification endoscopic observation using narrow band imaging (NBI)^17^ and enables the histological evaluation of UC via the determination of a Geboes score (GS).^18^ An AI-assisted diagnosis of histological healing, based on a GS < 3.1, may reduce unnecessary biopsies; however, there are no published reports regarding the clinical use of the AI system in patients with UC. This study compared the diagnostic performance for histological healing of the AI-assisted EndoBRAIN-UC system with that of conventional light non-magnifying endoscopic evaluations in patients with UC.

## Materials and Methods

This retrospective study was conducted at Tokyo Women’s Medical University from June to November 2021. Consecutive patients who met the diagnostic criteria for UC in Japan^1^ and underwent a total colonoscopy in a laboratory equipped with an ultra-magnifying endoscope were included in this study. Therefore, patients in the non-remitting phase were included. However, patients with severe symptomatic UC were excluded due to the potential physical burden of total colonoscope observation and the extended examination time. Patients who did not undergo a biopsy at the time of AI-assisted diagnosis were also excluded from the study. Non-magnified observations using white light were used to determine an MES diagnosis. Simultaneously, the AI system was used to diagnose the same site at which the MES diagnosis was obtained, and a biopsy was also performed. The sensitivity, specificity, positive predictive value (PPV), negative predictive value (NPV), and accuracy of the MES- and AI-assisted diagnoses were calculated using the biopsy pathology results as the gold standard. The results of the AI-assisted diagnosis for each MES value (0, 1, 2, and 3) were compared with the biopsy pathology results.

### MES Diagnoses

All patients underwent colonoscopy using the usual preparation of bowel-cleansing agents and an Olympus CF-H290ECI (Olympus) colonoscopy device. The colonoscopies and MES diagnoses were performed by 3 physicians with at least 10 years of endoscopic experience in patients with UC. The MES was agreed upon by the endoscopists using images of the biopsy site after colonoscopy. Discrepancies were resolved via discussion. MES0 was defined as mucosa in endoscopic remission.

### AI-Assisted Diagnoses

Each AI-assisted diagnosis was performed at the same site as the MES diagnosis using conventional endoscopy. The AI system was connected directly to an endoscopic system (EVIS LUCERA ELITE, Olympus). The latest version of the AI system (EndoBRAIN-UC) was recently approved in Japan, and each AI-assisted diagnosis was performed using the ultra-magnification function of the Olympus CF-H290ECI, a scope with a 12.8-mm-dia. tip, which provides a maximum magnification of 520×.

The NBI observation mode was used for the colonic mucosa in which the inflammatory activity was evaluated. The endoscope was then set to the maximum magnification (520×), and an ultra-magnified endoscopic image was acquired. When the image was captured, the program automatically analyzed the image, and the results of the analysis were displayed on a computer screen. The predicted output of the 2 categories (“Active” and “Healing”) and their probabilities (50% to 99%) were displayed on the endoscope monitor. If the probability of both categories was < 70%, the AI system reported “Low Confidence” rather than a specific prediction. If the AI-assisted diagnosis predicted a histological evaluation of GS < 3.1, the system reported “Healing,” and if a GS ≥ 3.1 was predicted, the system reported “Active” (Supplementary Figure S1). At least 5 AI diagnoses were made for the same site, and the most repeated result was used as the final diagnosis.

### Pathological Diagnoses

Each pathological diagnosis was made using biopsies of the same mucosal surface imaged for the MES- and AI-assisted diagnoses. The pathological diagnosis was made by a single pathologist using prepared hematoxylin and eosin-stained specimens, with a final agreement with a second pathologist. The GS was used for pathological diagnoses (Supplementary Table S1). The GS subdivides grades according to morphological changes in the mucosal tissue and inflammatory cell infiltration. In this study, histological healing was defined as a GS < 3.1 with no histological erosions, ulcers, or crypt neutrophil infiltration.

### Statistical Analyses

All data are expressed as median and interquartile range (IQR). The sensitivity, specificity, PPV, NPV, accuracy, and precision of the diagnostic methods were determined using Fisher’s test and a 2 × 2 table. JMP statistical analysis software (version 16; SAS) was used for all analyses.

## Ethical Considerations

The study protocol was approved by the Institutional Ethics Review Committee of our hospital on January 7, 2023 (IRB number 2022-0123). Based on the retrospective nature of the study, all patients were offered the opportunity to refuse treatment. A public announcement was posted on our website on January 7, 2023, as approved by the ethics committee.

## Results

### Patient Characteristics

Fifty-two patients with UC were included in the study (Table 1). The median disease duration was 13 years, and 73.1% of patients had a Montreal classification of E2 or E3. The median partial Mayo score was 0 (IQR: 0–0), and the median Lichtiger index was 3 (IQR: 3–3) at the time of colonoscopy. The median fecal immunochemical test result was 0 ng/mL (IQR: 0–75 ng/mL), and the median leucine-rich alpha 2 glycoprotein value was 12.7 (IQR: 10.1–13.7). Therefore, most of the patients were in clinical remission. The maximum MES value was MES0 in 16 (30.8%) patients, MES1in 16 (30.8%) patients, and MES2 in 20 (38.5%) patients. No patients had MES3. A total of 191 colonic mucosa sites were evaluated histologically, including 159 (83.2%) sites with a GS < 3.1. MES0 was detected at 129 (67.5%) sites, MES1 at 34 (17.8%) sites, and MES2 at 28 (14.7%) sites. The AI system reported 120 sites as healing and 71 as active (Table 2).

**Table 1.** Patient characteristics.

n		52 (%)
Males		32 (61.5)
Age, y		47.1 (37.9–56)
Disease duration, y		13 (7.4–20.7)
Montreal classification: E1/E2/E3		14 (26.9)/18 (34.6)/20 (38.5)
Partial Mayo score		0 (0–0)
Lichtiger index		3 (3–3)
Medication:		
5ASA		44 (84.6)
PSL		1 (1.9)
AZA		10 (19.2)
TOF		3 (5.8)
TAC		1 (1.9)
Anti-TNFα inhibitor		3 (5.8)
IL-12/23p40 inhibitor		3 (5.8)
Integrin inhibitor		2 (3.8)
Topical		19 (36.5)
WBC/μL		5960 (4485–6760)
Hb, g/dL		14 (12.9–14.9)
Plt × 10^4^/μL		24.5 (21.7–28.4)
Alb, g/dL		4.4 (4.2–4.6)
CRP, mg/dL		0.08 (0.05–0.2)
LRG, μg/mL		12.7 (10.1–13.7)
Fecal immunochemical test, ng/mL		0 (0–75)
Endoscopic diagnosis		Total *n* = 52 (%)
Colonoscopy score	MES	1 (0–2)
	UCEIS	3 (0–6)
Maximum MES in total colon:		
MES 0		16 (30.8)
MES 1		16 (30.8)
MES 2		20 (38.5)
MES 3		0(0)

Data are presented as median (interquartile range) or *n* (%).

Abbreviations: 5ASA, 5-aminosalicylic acid; Alb, albumin; AZA, azathioprine; CRP, C-reactive protein; Hb, hemoglobin; IL, interleukin; LRG: leucine-rich alpha 2 glycoprotein, MES, Mayo endoscopic score; Plt, platelets; PSL, prednisolone; TAC, tacrolimus; TNF, tumor necrosis factor; TOF, tofacitinib; UC, ulcerative colitis endoscopic index of severity; WBC, white blood cell.

**Table 2.** Histological characteristics.

AI-assisted diagnosis	
No. of evaluation points	191
AI healing/active	120 (62.8)/71 (37.2)
Histological evaluation	
No. of evaluation points	191
Biopsy points of MES: 0/1/2	129 (67.5)/34 (17.8)/28 (14.7)
Geboes score <3.1/no. of evaluation points	159 (83.2)

Data are presented as *n* (%).

Abbreviations: AI, artificial intelligence; MES, Mayo endoscopic score.

### Diagnostic Yields of MES and AI for Pathology

MES had a sensitivity of 79.2%, specificity of 90.6%, PPV of 97.7%, NPV of 46.8%, and accuracy of 81.2% for the diagnosis of GS < 3.1. The AI system had a sensitivity of 74.2%, specificity of 93.8%, PPV of 98.3%, NPV of 42.3%, and accuracy of 77.5% for the diagnosis of GS < 3.1 (Tables 3 and 4).

**Table 3.** Comparison of Geboes score based on AI diagnosis and MES diagnosis.

	GS < 3.1	GS ≥ 3.1	Total		GS < 3.1	GS ≥ 3.1	Total
Healing	118	2	120	MES 0	126	3	129
Active	41	30	71	MES ≥ 1	33	29	62
Total	159	32	191	Total	159	32	191

**Table 4.** Diagnostic performance for Geboes score <3.1.

	*P* value	Sensitivity 95%CI	Specificity 95%CI	PPV 95%CI	NPV 5%CI	Accuracy 95%CI
MES diagnosis: MES0	<.0001	79.2 (76.6–80.5)	90.6 (77.4–96.7)	97.7 (94.4–99.2)	46.8 (39.9–49.1)	81.2 (76.7–83.2)
AI-assisted diagnosis: Healing	<.0001	74.2 (71.7–75.1)	93.8 (81.1–98.3)	98.3 (95–99.5)	42.3 (36.6–44.3)	77.5 (73.3–79)

Abbreviations: AI, artificial intelligence; CI, confidence interval; MES, Mayo endoscopic score; NPV, negative predictive value; PPV, positive predictive value.

### Comparison of AI-Assisted Diagnosis and Pathology for Each MES Value

**Figure 1.**
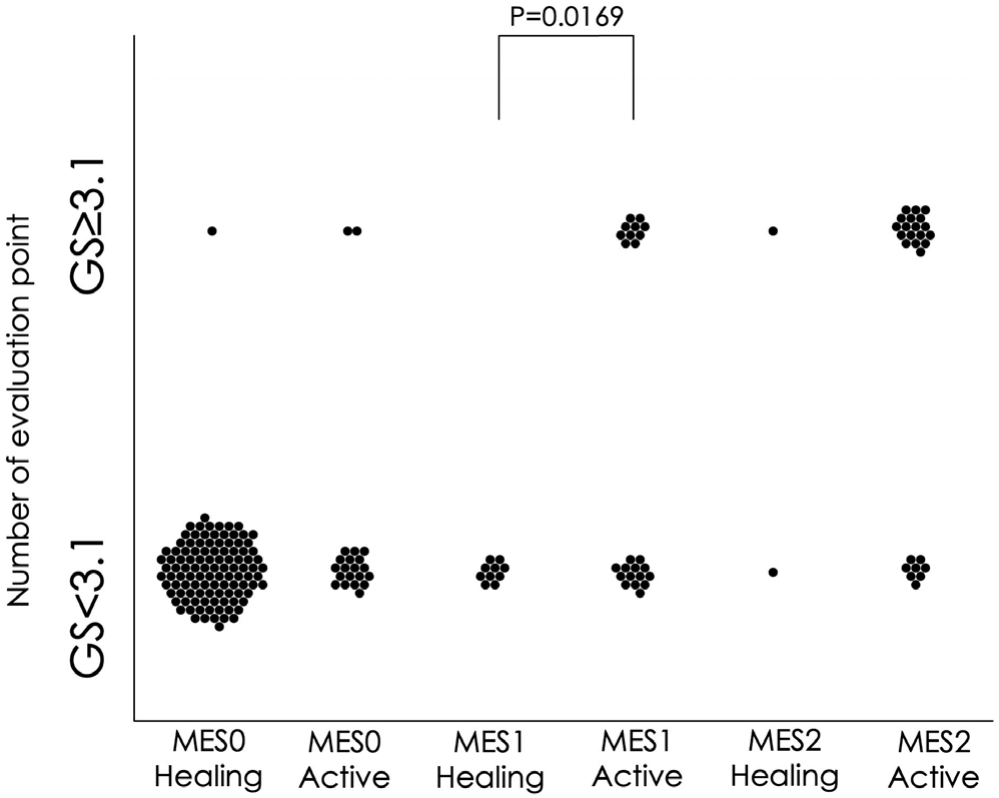
Comparison of Geboes score based on artificial intelligence (AI) diagnosis and Mayo endoscopic score (MES). The percentage of lesions with a Geboes score (GS) < 3.1 was significantly higher when the AI-assisted diagnosis was healing in MES1 lesions. In MES0 and MES2, the percentage of GS < 3.1 was not significantly different between the AI-assisted diagnoses.

For all MES values, there were both “Healing” and “Active” decisions based on AI-assisted diagnosis. Among the MES0 lesions, the AI system diagnosed 83.7% as the Healing decision. In MES0, there was no significant difference in the percentage of GS < 3.1 in the Healing and the Active decisions based on AI-assisted diagnosis. Among the MES2 lesions, the AI system diagnosed 92.9% as Active decision. In MES2, there was also no significant difference in the percentage of GS < 3.1 regardless of the result of AI-assisted diagnosis. Among the MES1 lesions, 29.4% were classified as the Healing decision and 70.6% as the Active decision. In MES1, the Healing decision with AI-assisted diagnosis identified significantly more GS < 3.1 than did the Active decision with AI-assisted diagnosis. (*P* = .0169) (Table 5 and Figure 1).

**Table 5.** AI diagnostic performance and proportion of Geboes <3.1 in each endoscopic assessment score.

MES diagnosis	AI-assisted diagnosis	No. of samples	GS < 3.1 (%)	*P* value
MES0	Healing	108	107 (99.1)	.0687
MES0	Active	21	19 (90.5)	
MES1	Healing	10	10 (100)	.0169
MES1	Active	24	14 (58.3)	
MES2	Healing	2	1 (50)	1.0000
MES2	Active	26	8 (30.8)	

Data are presented as the number (%) of biopsy points.

Abbreviations: AI, artificial intelligence; GS, Geboes score; MES, Mayo endoscopic score.

## Discussion

The EndoBRAIN-UC system became commercially available following a study by Maeda et al.^18^ regarding the use of AI in the management of UC. This system detects the GS score via a histological evaluation of UC based on endoscopy that is capable of ultra-magnified observation. Maeda et al. reported different relapse rates at 1 year for lesions reported as active or healing by the AI system.^19^ However, the previous study was conducted in a research and development facility; no reports of the real-world clinical utility of ultra-magnified endoscopic observation of UC using commercially available AI systems have been reported.

Histological evaluations based on the capillary structure of the mucosa obtained using automated evaluation systems have been reported,^20^ though no studies have demonstrated the usefulness of the system in actual clinical practice. In addition, studies regarding the use of AI-assisted diagnoses with non-magnified white light observation to determine the MES have been reported, though these devices are not commercially available.^21–23^ This study compares the diagnostic yield of the commercially available EndoBRAIN-UC system with that of MES in a real-world clinical setting.

In this study, the diagnostic yield for a GS < 3.1 was similar between MES (using white-light observation) and the AI. The diagnostic yield of the AI-assisted diagnosis was equivalent to that in the report by Maeda et al.^18^ with the exception of the NPV, confirming the high reproducibility of the AI-assisted diagnosis in clinical settings.

The NPV in the current study was lower than previously reported NPVs as this investigation was conducted in a real-world setting and fewer MES ≥ 2 specimens were endoscopically classified as inflammatory. Additionally, the results of the AI system used in this study do not fully reflect the pathological results as the GS is based on various factors including inflammatory cell infiltration and crypt destruction.^13^

Among the present 52 cases, 83.7% of the MES0 cases were judged to be Healing by the AI-assisted diagnosis, and 97.7% of the MES0 cases were GS < 3.1 by histological diagnosis. On the other hand, 92.9% of the MES2 cases were also judged as Active in the AI-assisted diagnosis, and only 32.1% of the MES2 cases were judged as GS < 3.1 by histological diagnosis. Therefore, an AI-assisted diagnosis may not be necessary when MES0 or MES2 can be clearly determined using unmagnified white light observation. Unnecessary ultra-magnification should be avoided as it is a more technical procedure that may increase the examination time compared to conventional endoscopy.^19^ Although an endoscopic diagnosis involves subjectivity and may lead to divided judgment, MES0 and MES2 are unlikely to be confused.^8,11,12^ In addition, an AI system that provides MES classifications using unmagnified white light may be commercially available in the future.^22,23^

**Figure 2.**
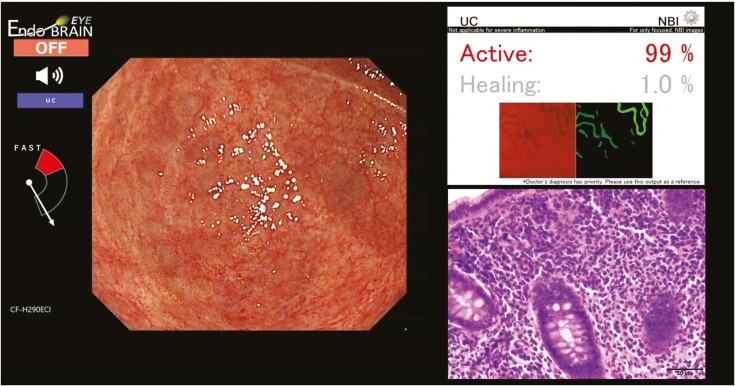
AI-assisted diagnosis in an MES1 lesion. In a patient with non-active ulcerative colitis, the cecum had erythematous mucosa with atrophic regenerating vessels. Endoscopically, the diagnosis was MES1, though the artificial intelligence-assisted diagnosis was active. Pathology revealed a Geboes score of 3.1 with neutrophilic infiltration.

Different prognoses for subsequent relapses have been reported for MES0 and MES1.^9,24,25^ However, MES1 lesions often do not relapse. Although the presence of histological inflammation plays a role in relapse,^13–16^ there may be intervening histological differences in the mucosa classified as MES1 (Figure 2). In the present study, the AI system reported a GS < 3.1 in MES1 lesions as healing, suggesting that this AI-based diagnosis may help determine differences in histological inflammation and the subsequent risk of relapse.

Histological determinations are conducted to evaluate inflammation and to diagnose neoplastic lesions. However, in cases where the purpose of biopsy is to evaluate inflammation, tissue sampling can be reduced if inactive mucosa can be identified without biopsy. The use of AI-assisted diagnosis may reduce unnecessary biopsies for the diagnosis of MES1, although prospective studies with relapse as an outcome are required to test this possibility.

This study has several limitations. This was a single-center, retrospective analysis of a small number of patients. This approach reveals the degree of inflammation but does not contribute to the detection of dysplasia. It is also unclear whether treatment interventions affect AI-assisted diagnoses, and further studies are required. In addition, ultra-magnification requires technical proficiency, which may have affected the results. However, the AI-assisted diagnoses with ultra-magnification were all performed by the same experienced endoscopists. The AI-assisted diagnostic protocol also included obtaining multiple images from the same site, and the most reproducible results were used. This reduces the technical influence as much as possible.

## Conclusion

In conclusion, MES and AI-assisted diagnoses have similar diagnostic yields for a GS < 3.1. An AI-based diagnosis of MES1 may reduce the need for biopsies for histologic examination.

## Supplementary Material

otae005_suppl_Supplementary_Figures_S1

otae005_suppl_Supplementary_Tables_S1

## Data Availability

The data underlying this article will be shared upon reasonable request to the corresponding author.

## References

[1] Nakase H , Uchino M, Shinzaki S, et al. Evidence-based clinical practice guidelines for inflammatory bowel disease 2020. J Gastroenterol. 2021;56(6):489-526. 33885977 10.1007/s00535-021-01784-1 PMC8137635

[2] Murakami Y , Nishiwaki Y, Oba MS, et al. Estimated prevalence of ulcerative colitis and Crohn’s disease in Japan in 2014: an analysis of a nationwide survey. J Gastroenterol. 2019;54:1070-1077. 31309327 10.1007/s00535-019-01603-8

[3] Ardizzone S , Cassinotti A, Duca P, et al. Mucosal healing predicts late outcomes after the first course of corticosteroids for newly diagnosed ulcerative colitis. Clin Gastroenterol Hepatol. 2011;9:483-489.e3. 21195796 10.1016/j.cgh.2010.12.028

[4] Rutter M , Saunders B, Wilkinson K, et al. Severity of inflammation is a risk factor for colorectal neoplasia in ulcerative colitis. Gastroenterology. 2004;126:451-459. 14762782 10.1053/j.gastro.2003.11.010

[5] Neurath MF , Travis SPL. Mucosal healing in inflammatory bowel diseases: a systematic review. Gut. 2012;61:1619-1635. 22842618 10.1136/gutjnl-2012-302830

[6] Turner D , Ricciuto A, Lewis A, et al.; International Organization for the Study of IBD. STRIDE-II: an update on the selecting therapeutic targets in inflammatory bowel disease (STRIDE) initiative of the international organization for the study of IBD (IOIBD): determining therapeutic goals for treat-to-target strategies in IBD. Gastroenterology. 2021;160:1570-1583. 33359090 10.1053/j.gastro.2020.12.031

[7] Schroeder KW , Tremaine WJ, Ilstrup DM. Coated oral 5-aminosalicylic acid therapy for mildly to moderately active ulcerative colitis. N Engl J Med. 1987;317:1625-1629. 3317057 10.1056/NEJM198712243172603

[8] Rutgeerts P , Sandborn WJ, Feagan BG, et al. Infliximab for induction and maintenance therapy for ulcerative colitis. N Engl J Med. 2005;353:2462-2476. 16339095 10.1056/NEJMoa050516

[9] Acosta MB , Vallejo N, Iglesia D, et al. Evaluation of the risk of relapse in ulcerative colitis according to the degree of mucosal healing (Mayo 0 vs 1): a longitudinal cohort study. J Crohns Colitis. 2016;10:13-19. 26351390 10.1093/ecco-jcc/jjv158

[10] Vuitton L , Peyrin-Biroulet L, Colombel JF, et al. Defining endoscopic response and remission in ulcerative colitis clinical trials: an international consensus. Aliment Pharmacol Ther. 2017;45:801-813. 28112419 10.1111/apt.13948

[11] de Lange T , Larsen S, Aabakken L. Inter-observer agreement in the assessment of endoscopic findings in ulcerative colitis. BMC Gastroenterol. 2004;4:9. 15149550 10.1186/1471-230X-4-9 PMC434504

[12] Osada T , Ohkusa T, Yokoyama T, et al. Comparison of several activity indices for the evaluation of endoscopic activity in UC: inter- and intraobserver consistency. Inflamm Bowel Dis. 2010;16:192-197. 19575359 10.1002/ibd.21000

[13] Geboes K , Riddell R, Ost A, Jensfelt B, Persson T, Löfberg R. A reproducible grading scale for histological assessment of inflammation in ulcerative colitis. Gut. 2000;47:404-409. 10940279 10.1136/gut.47.3.404 PMC1728046

[14] Bryant RV , Burger DC, Delo J, et al. Beyond endoscopic mucosal healing in UC: histological remission better predicts corticosteroid use and hospitalisation over 6 years of follow-up. Gut. 2016;65:408-414. 25986946 10.1136/gutjnl-2015-309598

[15] Park S , Abdi T, Gentry M, Laine L. Histological disease activity as a predictor of clinical relapse among patients with ulcerative colitis: systematic review and meta-analysis. Am J Gastroenterol. 2016;111:1692-1701. 27725645 10.1038/ajg.2016.418

[16] Fluxá D , Simian D, Flores L, et al. Clinical, endoscopic and histological correlation and measures of association in ulcerative colitis. J Dig Dis. 2017;18:634-641. 28949435 10.1111/1751-2980.12546

[17] Maeda Y , Ohtsuka K, Kudo SE, et al. Endocytoscopic narrow-band imaging efficiency for evaluation of inflammatory activity in ulcerative colitis. World J Gastroenterol. 2015;21:2108-2115. 25717245 10.3748/wjg.v21.i7.2108 PMC4326147

[18] Maeda Y , Kudo SE, Mori Y, et al. Fully automated diagnostic system with artificial intelligence using endocytoscopy to identify the presence of histologic inflammation associated with ulcerative colitis (with video). Gastrointest Endosc. 2019;89:408-415. 30268542 10.1016/j.gie.2018.09.024

[19] Maeda Y , Kudo S, Ogata N, et al. Evaluation in real-time use of artificial intelligence during colonoscopy to predict relapse of ulcerative colitis: a prospective study. Gastrointest Endosc. 2022;95:747-756.e2. 34695422 10.1016/j.gie.2021.10.019

[20] Bossuyt P , Hertogh GD, Eelbode T, Vermeire S, Bisschops R. Computer-aided diagnosis with monochromatic light endoscopy for scoring histologic remission in ulcerative colitis. Gastroenterology. 2021;160:23-25. 33058863 10.1053/j.gastro.2020.09.053

[21] Takenaka K , Ohtsuka K, Fujii T, et al. Development and validation of a deep neural network for accurate evaluation of endoscopic images from patients with ulcerative colitis. Gastroenterology. 2020;158:2150-2157. 32060000 10.1053/j.gastro.2020.02.012

[22] Takenaka K , Fujii T, Kawamoto A, et al. Deep neural network for video colonoscopy of ulcerative colitis: a cross-sectional study. Lancet Gastroenterol Hepatol. 2022;7:230-237. 34856196 10.1016/S2468-1253(21)00372-1

[23] Bossuyt P , Nakase H, Vermeire S, et al. Automatic, computer-aided determination of endoscopic and histological inflammation in patients with mild to moderate ulcerative colitis based on red density. Gut. 2020;69:1778-1786. 31915237 10.1136/gutjnl-2019-320056

[24] Yokoyama K , Kobayashi K, Mukae M, Sada M, Koizumi W. Clinical study of the relation between mucosal healing and long-term outcomes in ulcerative colitis. Gastroenterol Res Pract. 2013;2013:192794. 23762033 10.1155/2013/192794 PMC3665176

[25] Mazzuoli S , Guglielmi FW, Antonelli E, Salemme M, Bassotti G, Villanacci V. Definition and evaluation of mucosal healing in clinical practice. Dig Liver Dis. 2013;45:969-977. 23932331 10.1016/j.dld.2013.06.010

