# Supplementary material for: Comparison of Endoscopic and Artificial Intelligence Diagnoses for Predicting the Histological Healing of Ulcerative Colitis in a Real-World Clinical Setting
Source: Crohns Colitis 360. 2024 Jan 20;6(1):otae005. doi: 10.1093/crocol/otae005 (PMC10901431; doi:10.1093/crocol/otae005)
Supplement: otae005_suppl_Supplementary_Figures_S1 [file otae005_suppl_supplementary_figures_s1.zip › otae005/Figure S1.docx]

**Supplementary Material**

**Figure S1. AI-assisted diagnosis.** When the AI-assisted diagnosis predicts a histological evaluation of a Geboes score (GS)<3.1, it reports “healing.” When a GS≥3.1 is predicted, the system reports “active.”
