# Supplementary figures and images for: Comparison of Endoscopic and Artificial Intelligence Diagnoses for Predicting the Histological Healing of Ulcerative Colitis in a Real-World Clinical Setting
Source: Crohns Colitis 360. 2024 Jan 20;6(1):otae005. doi: 10.1093/crocol/otae005 (PMC10901431; doi:10.1093/crocol/otae005)

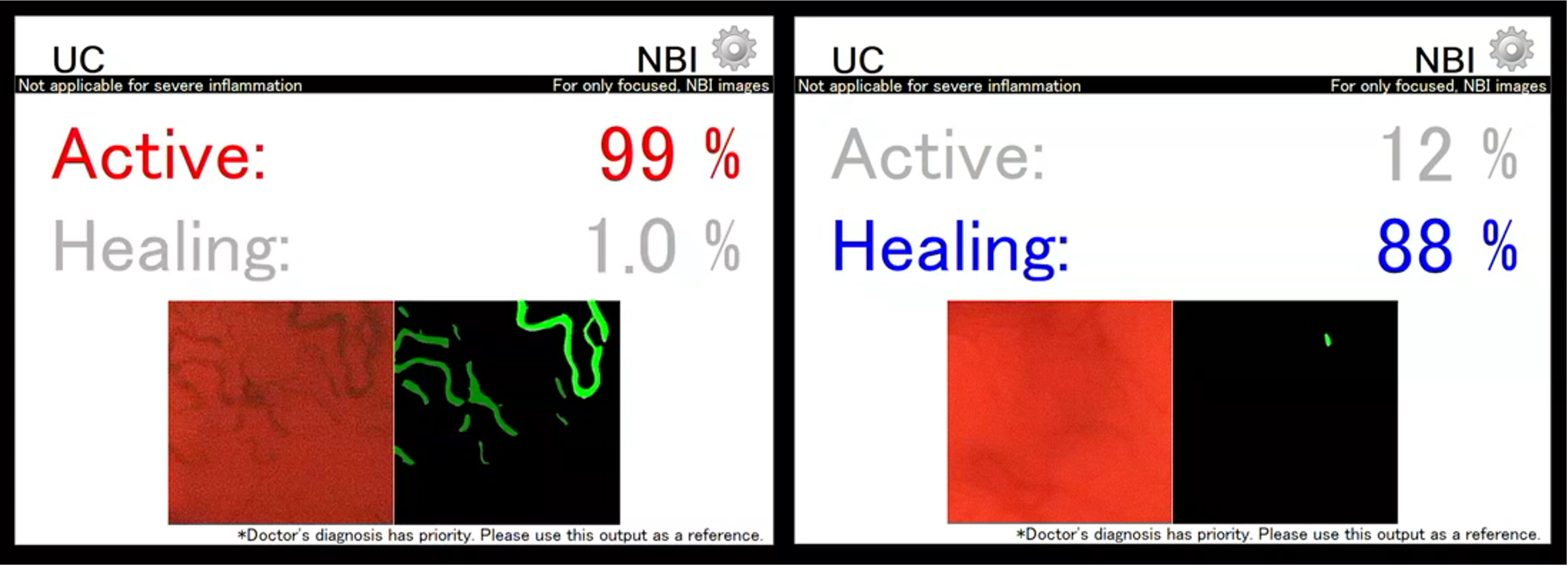

Supplement: otae005_suppl_Supplementary_Figures_S1 [file otae005_suppl_supplementary_figures_s1.zip › otae005/UC-AI-retro-CC360-supplementary-Figure.tiff]
