# Supplementary material for: Comparison of Endoscopic and Artificial Intelligence Diagnoses for Predicting the Histological Healing of Ulcerative Colitis in a Real-World Clinical Setting
Source: Crohns Colitis 360. 2024 Jan 20;6(1):otae005. doi: 10.1093/crocol/otae005 (PMC10901431; doi:10.1093/crocol/otae005)
Supplement: otae005_suppl_Supplementary_Tables_S1 [file otae005_suppl_supplementary_tables_s1.docx]

**Supplementary Table S1.** Geboes Score

| Structural (architectural changes) | Grade 0 |
| --- | --- |
| No abnormality | 0.0 |
| Mild abnormality | 0.1 |
| Mild or moderate diffuse or multifocal abnormalities | 0.2 |
| Severe diffuse or multifocal abnormalities | 0.3 |
| Chronic inﬂammatory inﬁltrate | Grade 1 |
| No increase | 1.0 |
| Mild but unequivocal increase | 1.1 |
| Moderate increase | 1.2 |
| Marked increase | 1.3 |
| Lamina propria eosinophils | Grade 2A |
| No increase | 2A.0 |
| Mild but unequivocal increase | 2A.1 |
| Moderate increase | 2A.2 |
| Marked increase | 2A.3 |
| Lamina propria neutrophils | Grade 2B |
| No increase | 2B.0 |
| Mild but unequivocal increase | 2B.1 |
| Moderate increase | 2B.2 |
| Marked increase | 2B.3 |
| Neutrophils in epithelium | Grade 3 |
| None | 3.0 |
| < 5 % Crypts involved | 3.1 |
| < 50 % Crypts involved | 3.2 |
| > 50 % Crypts involved | 3.3 |
| Crypt destruction | Grade 4 |
| None | 4.0 |
| Probable - local excess of neutrophils in part of crypt | 4.1 |
| Probable - marked attenuation | 4.2 |
| Unequivocal crypt destruction | 4.3 |
| Erosion or ulceration | Grade 5 |
| No erosion, ulceration or granulation tissue | 5.0 |
| Recovering epithelium + adjacent inﬂammation | 5.1 |
| Probable erosion focally stripped | 5.2 |
| Unequivocal erosion | 5.3 |
| Ulcer or granulation tissue | 5.4 |
